# US cat caregivers’ attitudes on veterinary video telemedicine

**DOI:** 10.1177/1098612X241249623

**Published:** 2024-08-09

**Authors:** Sooyoung Lee, Grace Boone, Ashley Bidgoli, Joshua Di Bernardo, Carly M Moody

**Affiliations:** Department of Animal Science, University of California, Davis, CA, USA

**Keywords:** Video telemedicine, companion animals, virtual care, animal welfare, cat stress, cat owner, behavior

## Abstract

**Objectives:**

Many cats do not see a veterinarian on an annual basis, and their caregivers face many barriers to accessing veterinary care. A potential solution to overcome some of these barriers is video telemedicine. Thus, the aim of this study was to understand companion cat caregivers’ perceptions of using veterinary video telemedicine with their cats.

**Methods:**

An online quantitative questionnaire was used to survey US cat caregivers on their experiences of and attitudes to using video telemedicine with their cats. Participants were required to reside in the USA, be the primary caregiver of at least one cat and be aged 18 years or older.

**Results:**

The majority (97.3%) of the 1254 respondents indicated they had never used a video telemedicine appointment with their cat(s) before; however, most (85.7%) indicated they were very or somewhat interested in using video telemedicine with their cat. Overall, caregivers perceived video telemedicine visits as less stressful for themselves (*P* <0.0002) and their cats (*P* <0.0001), and as increasing their access to veterinary care (*P* <0.0001) compared with in-clinic visits. Participants also indicated they would prefer a video telemedicine appointment over an in-clinic appointment for most cat behavioral concerns but preferred in-clinic appointments for most health concerns. Most respondents (51.3%) indicated they would be willing to pay a little less for a telemedicine appointment than an in-clinic visit.

**Conclusions and relevance:**

Cat caregivers represent an important population that could benefit from the implementation of video telemedicine into veterinary care. Our results suggest many US cat caregivers indicate an interest and willingness to pay for video telemedicine visits to increase their access to veterinary care and reduce stress associated with veterinary visits for both themselves and their cats. Caregivers showed more interest in using video telemedicine for behavioral concerns than health concerns.

## Introduction

Cats are popular companion animals, with an estimated 60 million pet cats in the USA.^1^ However, research has shown that many cat caregivers have a reduced willingness to bring their cat to the veterinary clinic for several reasons, including cats’ aversion towards carriers and travel, and caregivers’ perception of cat stress during clinic visits.^2–5^ Further, caregiver-related factors, such as distance to clinics, access to transportation, living with disability and socioeconomic status, can make access to care more difficult.^6–8^ The barriers caregivers face to accessing care is concerning, given that all cats should receive routine care to prevent, detect and manage health and behavior issues. With the advancement of technology, veterinary video telemedicine has emerged as a possible solution to help ease cat and owner stress related to clinic visits, and overcome other barriers related to accessing veterinary care, such as low geographic distribution of veterinary clinics in rural and low-income areas.^9,10^ Telemedicine is defined as ‘the use of a tool to exchange information about a patient’s clinical health status electronically from one site to another’^11^ and may be used to enhance traditional in-clinic care, not replace it. To use telemedicine in veterinary healthcare, a veterinarian–client–patient relationship (VCPR) must be established before use, which may involve an in-person appointment; however, in some regions and situations this may be established virtually.^11^ The VCPR is an important aspect of veterinary care, as it is the basis of the relationship between the veterinarian, client and patient, which is utilized during diagnosis and treatment. After a VCPR has been established, remote practice of veterinary medicine is permitted, although details of use vary with regional laws and regulations.

Telemedicine may take a variety of forms, such as phone calls or email communications, or synchronous visits conducted via video conferencing platforms. Video telemedicine involves video communication and may include a limited clinical assessment of the patient in addition to collecting history from the caregiver. This could include observing parameters such as the animal’s gait, skin, coat or behavior. Recommendations for incorporating video telemedicine into veterinary care suggest virtual visits as an alternative way to conduct a variety of appointment types, including behavioral evaluations, postoperative rechecks, nutritional cases, new client meet and greets, puppy/kitten information sessions, triage and for the management and monitoring of chronic conditions.^12,13^ However, for video telemedicine to be a viable strategy for improving access to veterinary care, healthcare providers must be willing to use this mode of healthcare provision, and provide it to pet owners.

In the past few years, research shows that use of veterinary video telemedicine has been increasing, particularly since the start of the COVID-19 pandemic;^14–16^ however, data suggest that the incorporation of video telemedicine into practice may not yet be widespread. For example, a study examining veterinarians’ use of telemedicine in Germany reported that in-person and telephone communication were more common than video calls.^17^ Veterinarians in the USA reported occasional telehealth and telemedicine use, but participants in these studies showed a lack of knowledge regarding the difference between the two terms.^15,18^ Potential reasons for the limited use of telemedicine by veterinarians include a desire for clear professional guidelines on use,^17^ belief there should be limits on use,^19^ attitudes that telemedical care is more appropriate for existing patients^17,19^ and concerns about whether video telemedicine has enhanced or damaged the VCPR.^20,21^ Veterinarians in a recent survey reported less confidence in their ability to reach a diagnosis during a virtual consultation compared with an in-person examination, and indicated that it may be more challenging to express empathy and build rapport virtually.^22^ Research has also highlighted concerns about legality regarding licensing, establishing a VCPR virtually and the risk of personal information leakage, which increase veterinarians’ and clients’ hesitancy to utilize online platforms for veterinary appointments.^12,14,23^

Despite these concerns, pilot work examining the use of telehealth by veterinary students with underserved populations suggested that both students and clients viewed the experience very positively, and the program increased access to care by reducing cost and transportation barriers.^24^ In addition, survey research suggested that many veterinarians recognized telemedicine as a way to increase access to veterinary care,^19^ and some indicated they intended to sustain their use of telemedicine in the future.^15,18^ A survey of pet owners using a popular telemedicine website in the USA suggested that many telemedicine visits concluded with a recommendation to follow-up with a traditional care veterinarian; participants largely completed these follow-ups, and felt better informed about their pet’s illness and more able to effectively communicate with their veterinarian after the telemedicine visit.^21^ In addition, most participants stated that their traditional care veterinarian agreed with the veterinary telemedicine expert’s recommendation.^21^ Other research has examined pet caregivers’ attitudes to the use of video telemedicine. For example, a study of pet caregivers in Germany suggested many caregivers perceived veterinary telemedicine as beneficial, although most had not yet utilized it.^25^ It is not clear if the lack of use was because their primary care veterinarian had not offered telemedicine as an option or because they had not sought veterinary care for their pet. Research also suggests that pet caregivers would use alternative ways of communicating with their veterinary clinic if they were offered, such as text or video.^21^ Caregivers further indicate they are willing to pay for telemedicine appointments^26,27^ and would pay more for one with their own veterinarian.^26^ However, some data suggest that not all caregivers are willing to use veterinary telemedicine because of perceptions of insufficient treatment options and lack of a personalized experience.^25^

Video telemedicine could be particularly useful for cats, given fewer cats than dogs receive veterinary care each year, and it is well known that clinic visits may result in negative experiences for cats and their owners.^2–5,28^ The use of video telemedicine appointments would allow cats to remain at home, reducing their exposure to carriers, travel, new people, new animals and a new environment. In addition, keeping the cat in the home may be beneficial for seeing its normal behavioral repertoire, which is commonly impacted by a clinic environment. Some research has assessed pet caregiver attitudes towards using veterinary telemedicine, although it lacks a focus on companion cats, which are a special population that may benefit from this technology. One study has focused on cats and their caregivers, examining cat responses and caregiver attitudes after participating in video telemedicine and in-person appointments for a mock spay recheck.^27^ In this study, cats showed reduced negative behavioral and physiological responses during video telemedicine appointments compared with in-clinic appointments, suggesting virtual veterinary care may be particularly beneficial for cats that show more fear or aggression during in-clinic visits. In addition, after undertaking both appointment types, participant caregivers indicated that video telemedicine improved their access to veterinary care and reduced their stress and their cat’s stress compared with in-clinic visits.^27^ Although these results are promising, this research was conducted on a small sample of cats and caregivers (n = 30), and there is a need to assess cat caregiver attitudes on a larger scale for greater generalizability. Thus, the aim of this research was to explore US companion cat caregivers’: (1) interest in using video telemedicine to access veterinary care for their cat; (2) perceptions of the impact of video telemedicine appointments on the accessibility of veterinary care compared with in-clinic visits; (3) perceptions of their own stress levels and their cat’s stress levels when accessing veterinary care using video telemedicine vs in-clinic appointments; and (4) appointment preferences (video telemedicine vs in-clinic) for a variety of cat health and behavior concerns. We predicted that cat caregiver interest in using veterinary video telemedicine for accessing care would depend on caregiver comfort with technology, willingness to pay, length of transportation time to their veterinarian, perceived stress reduction for themselves and their cat, as well as a perception of improved access to care. We also predicted that most respondents would indicate that video telemedicine appointments would increase their access to veterinary care and reduce their own and their cat’s stress levels compared with in-clinic appointments. Finally, we predicted that respondents would prefer video telemedicine appointments for health and behavior concerns that do not rely on a physical examination, such as weight management and destructive behaviors, and prefer in-clinic appointments for concerns requiring comprehensive physical examinations and diagnostics, such as dental disease and gastrointestinal disorders.

## Materials and methods

This study was reviewed by the University of California Davis Institutional Review Board (number 1883274). Participation in the study was voluntary and anonymous. Participants provided consent via an online consent form before accessing the questionnaire; they were asked to read the consent form and if they agreed to take part in the research, they could click on a button to access the questionnaire.

A questionnaire containing 54 questions was created using the online survey software Qualtrics (Qualtrics Software Company, Provo, Utah, USA). Participants were required to reside in the USA, be the primary caregiver of at least one cat and be aged at least 18 years. The questionnaire was in English, and internet access was needed to complete the survey. Participants were recruited through snowball sampling on social media sites (eg, Facebook, Twitter, Instagram, LinkedIn) and through an article published by Gizmodo (https://shorturl.at/abV39). Data collection took place between 19 October and 6 November 2022.

The questionnaire contained seven sections: (1) inclusion/exclusion criteria (five questions); (2) human demographics (six questions); (3) owner experience: in-clinic (eight questions); (4) owner experience: telemedicine (17 questions); (5) opinions on in-clinic appointments (six questions); (6) opinions on telemedicine (nine questions); and (7) comparisons of in-clinic and telemedicine appointments, and an open-ended comment box (three questions). The questionnaire defined video telemedicine as providing healthcare and/or behavior care for a pet or person over a distance using video conferencing (eg, Zoom).

A copy of the questionnaire can be found in the supplementary material for this paper. In summary, the human demographic questions included gender, residing area, dependents (yes, no) and amount of experience with cats, not including as a cat owner. If participants stated that they worked with cats, they were asked a follow-up question about the capacity in which they worked with cats. Participants were also asked to place themselves on a socioeconomic ladder scale of 1–10 based on where they stood in their local communities, with 10 being on the top and 1 being on the bottom.

Questions about owner in-clinic experiences included when their last in-clinic visit with their cat(s) was, average travel time to their veterinary clinic and if their cat(s) needed sedating medication at home before an in-clinic visit or during an in-clinic examination. Participants were also asked to list the examination styles their clinic had offered during COVID-19 and the activities they performed to prepare their cat before a veterinary visit. Likert-scale questions asked how easy it was for veterinarians and veterinary staff to handle their cat(s), the ease of interacting with their cat(s) at home after an appointment and the caregiver’s satisfaction with in-clinic visits.

Participants were asked binary (yes/no) questions about their prior experience using video conferencing software platforms and using video telemedicine for their cat(s), other pets and themselves. If they answered ‘yes’ to any of these questions, they were asked which type of technology was utilized (phone, computer or both), the reason for the visit (their cat[s] and other pets only) and their satisfaction with their experience. Those who had used video telemedicine with their cat were asked to rate the ease (scale: easy to difficult) of handling their cat during the appointment, and ease (scale: easy to difficult) of interacting with their cat after the appointment. Participants who indicated they had not utilized video telemedicine for their cat were asked to rate their perceived ease (scale: easy to difficult) of handling their cat during a video telemedicine appointment, and ease (scale: easy to difficult) of interacting with their cat after the appointment.

All participants were asked to rank their comfort with using video conferencing software, and their access to technology, reliable internet, the applications/websites needed and camera positioning/set-up on a 5-point Likert scale. They were also asked to indicate the methods of electronic communication offered by their veterinary clinic. Binary (yes/no) questions were also included regarding whether they had ever asked their veterinarian about using video telemedicine for their pets, and whether their veterinarian or veterinary staff had mentioned using video telemedicine for their cat. If they answered ‘yes’ to both questions, participants were asked to rate the attitude of the veterinarian and/or veterinary staff regarding use of video telemedicine using a 5-point Likert scale.

Questions related to participants’ opinions of in-clinic and video telemedicine appointments included: attitudes on the appropriate frequency of veterinary visits for kittens, young adult, mature and senior cats; prices they would be willing to pay for a video telemedicine visit compared with a non-emergency in-clinic visit; and if they would be willing to submit videos or photos to the veterinarian to supplement in-clinic or video telemedicine visits. Likert-scale questions were also used to assess interest in using veterinary video telemedicine with their cat(s), how appointment type impacted their access to veterinary care, and the perceived helpfulness of both in-clinic and video telemedicine visits for the following: affording veterinary care; finding appointments that worked with their schedule; finding cats for the appointment; addressing transportation challenges; addressing disability challenges; diagnosing health and behavior conditions; and developing treatment plans for health and behavior problems. Participants indicated perceived stress levels for themselves and their cat during in-clinic and video telemedicine appointments on a 5-point visual analog scale to indicate stress level, where 1 was the most stressed and 5 was the least stressed (those who had never utilized video telemedicine were asked to anticipate stress levels).

Lastly, participants were asked to choose their preferred appointment type for a variety of cat health and behavior concerns.

### Statistical analyses

Descriptive statistics (frequencies and percentages) were produced using Jamovi (Jamovi Project) and statistical tests were conducted using SAS Studio v3.7 (SAS Institute). For all tests, *P* <0.05 was considered statistically significant.

Logistic regression was used to assess the relationship between interest in telemedicine (binary outcome: somewhat interested and very interested = yes; somewhat uninterested and very uninterested = no; neutral responses were not included as they were not of interest) and 20 explanatory factors predicted to impact interest including owner demographics, social factors and accessibility ratings, as well as past owner and cat experiences at the clinic. We aimed to create the most parsimonious model to extract meaningful results and reduce complexities, thus two-way analyses using a liberal *P* value (*P* <0.2) were used to assess which of the 20 variables to include in the model. The model was then built using a backwards selection strategy whereby variables were taken out of the model if a *P* value was <0.05; a stepwise strategy was followed to ensure no significant variables were missed. Predicted and plausible two-way interactions were also tested during the model-building process. Given that all the explanatory variables included in the model were categorical, model fit was based on evaluation of the two-way interaction terms. After model building, post-hoc pairwise comparisons were run. To reduce the potential for type 1 errors, Tukey’s adjustment was used when multiple comparisons included four or more pairs. Results are reported using odds ratios (ORs), 95% confidence intervals (CIs) and *P* values.

Wilcoxon signed-rank tests (related samples test) were used to examine differences between in-clinic vs video telemedicine ratings of perceived owner stress, cat stress and impact on accessibility. When analyzing perceived owner stress and cat stress, tests were run separately for those who had used video telemedicine with their cat and those who had not. The categorical scale response options were converted to a numerical scale, and the ‘not sure’ category was not included to avoid a zero value.

A multiway contingency table using a χ^2^ analysis was used to test for an association between age and willingness to pay.

## Results

A total of 1636 responses were recorded in Qualtrics. Incomplete responses, duplicate IP addresses and participants not meeting the inclusion criteria were excluded, leaving a total of 1254 participants.

### Participant demographics

Most respondents indicated they had one (34.1%) or two (36.4%) cats (Table 1). The US states with the highest representation among respondents in this survey were California (24.1%), Florida (4.7%), New York (4.5%), Washington (4.4%) and Pennsylvania (4.1%) (see supplementary material). The majority of respondents were aged 30–39 years (22.8%) or 40–49 years (21.2%), were women (79.9%) and lived in suburban areas (58.6%). Most respondents reported they were the primary caretaker for cats only (74.8%), although approximately one-fifth also cared for dogs (20.3%), and a small percentage cared for other small animals (8.7%), such as caged mammals, reptiles or birds. Most participants (81.0%) indicated they did not have dependents such as children or others that relied on them for care. Participants most frequently rated themselves at level 7 (26.0%), 6 (20.7%), 8 (15.9%) or 5 (15.1%) on the socioeconomic ladder. In relation to cat experience, over half (56.4%) had no volunteer or work experience. Of those who did work with cats (n = 547), many had experience working in an animal shelter (56.3%), being a pet sitter (38.8%), working with cats in some other capacity (22.7%) or as veterinary clinic staff (17.9%).

**Table 1:** Demographic and descriptive information for US cat caregiver questionnaire respondents (n = 1254)

Variable	Category	n (%)
Age (years)	18–29	132 (10.5)
	30–39	286 (22.8)
	40–49	266 (21.2)
	50–59	252 (20.1)
	60–69	212 (16.9)
	⩾70	98 (7.8)
	Prefer not to say, but over 18 years	8 (0.6)
Gender	Man	206 (16.4)
	Woman	1002 (79.9)
	Non-binary, third gender or other	33 (2.6)
	Prefer not to say	13 (1.0)
Type of residence	Rural (settled place outside of a city)	181 (14.4)
	Suburban (residential area on the outskirts of a city)	735 (58.6)
	Urban (city center or metropolis)	338 (27.0)
Level on socioeconomic ladder	1	9 (0.7)
	2	26 (2.1)
	3	66 (5.3)
	4	105 (8.4)
	5	189 (15.1)
	6	260 (20.7)
	7	326 (26.0)
	8	200 (15.9)
	9	58 (4.6)
	10	15 (1.2)
Have dependents	No	1016 (81.0)
	Yes	238 (19.0)
Number of cats (primary caretaker)	1	427 (34.1)
	2	457 (36.4)
	3	170 (13.6)
	4	63 (5.0)
	⩾5	137 (10.9)
Other pets (primary caretaker)*	No	938 (74.8)
	Yes, dogs	254 (20.3)
	Yes, other (ie, small caged mammals, caged reptiles, birds)	109 (8.7)
Previous cat experience (years)	None	707 (56.4)
	<1	168 (13.4)
	1–5	242 (19.3)
	6–10	65 (5.2)
	>10	72 (5.7)
Profession of cat experience	Groomer	17 (1.4)
	Behaviorist	14 (1.1)
	Pet sitter	212 (16.9)
	Animal shelter staff/volunteer	308 (24.6)
	Trainer	7 (0.8)
	Veterinary clinic staff	98 (7.8)
	Other	124 (9.9)

* Adds up to more than 1254 as respondents could select multiple options

### In-clinic experiences and perceptions

Most respondents indicated that their last veterinary visit had been within the past year (79.2%), that their cats did not receive veterinarian-prescribed calming or sedating medication before being taken to the clinic (75.6%) and their cat(s) never had to be sedated for an in-clinic examination (70.7%) (Table 2). Most caregivers (83.9%) reported a clinic travel time of less than 30 mins.

**Table 2:** Descriptive information on US cat caregivers’ (n = 1254) experiences and perceptions of in-clinic veterinary visits for their cat(s)

Variable	Category	n (%)
Last visit to veterinary clinic with their cat(s) (years)	Within past year	993 (79.2)
	1–2	173 (13.8)
	3–5	58 (4.6)
	>5	21 (1.7)
	Never	9 (0.7)
Veterinarian has prescribed calming or sedating medication to give their cat(s) before clinic visits	Yes	298 (23.8)
	No	948 (75.6)
	Not sure	8 (0.6)
Their cat(s) has to be sedated for in-clinic examination	Often	72 (5.7)
	Sometimes	128 (10.2)
	Rarely	113 (9.0)
	Never	887 (70.7)
	Not sure	54 (4.3)
Travel time to veterinary clinic	<30 mins	1052 (83.9)
	30–59 mins	186 (14.8)
	1–1.5 h	14 (1.1)
	>1.5 h	2 (0.2)
Preparing their cat for an in-clinic visit*	Take carrier out before appointment	683 (54.5)
	Cover carrier with a towel	306 (24.4)
	Leave carrier out all time	288 (23.0)
	Play soothing music in car	238 (19.0)
	Pheromone spray on carrier	183 (14.6)
	Other	214 (17.1)
	None of the above	208 (16.6)
Perceptions of veterinarian handling their cat(s)*	Very easy	554 (44.2)
	Somewhat easy	409 (32.6)
	Neither easy nor difficult	228 (18.2)
	Somewhat difficult	308 (24.6)
	Very difficult	157 (12.5)
	Not sure	29 (2.3)
Perceptions of veterinary staff handling their cat(s)*	Very easy	546 (43.5)
	Somewhat easy	415 (33.1)
	Neither easy nor difficult	220 (17.5)
	Somewhat difficult	297 (23.7)
	Very difficult	411 (32.8)
	Not sure	34 (2.7)
Interacting with cat(s) at home after clinic visit*	Very easy	663 (52.9)
	Somewhat easy	300 (23.9)
	Neither easy nor difficult	218 (17.4)
	Somewhat difficult	187 (14.9)
	Very difficult	56 (4.5)
	Not sure	28 (2.2)
Satisfaction with in-clinic veterinary visits for their cat(s)	Very satisfied	596 (47.5)
	Somewhat satisfied	445 (35.5)
	Neither satisfied nor dissatisfied	89 (7.1)
	Somewhat dissatisfied	103 (8.2)
	Very dissatisfied	21 (1.7)

* Adds up to more than 1254 as respondents could select multiple options

When asked about preparing their cat(s) for a clinic visit (respondents could choose all options that applied), most respondents indicated they took out their carrier before the appointment (54.5%), approximately one-quarter covered the carrier with a towel during travel (24.4%) and/or left the carrier out all the time (23.0%). During in-clinic veterinary visits, most participants indicated handling their cat(s) was very easy (veterinarian: 44.2%, veterinary staff: 43.5%) or somewhat easy (veterinarian: 32.6%, veterinary staff: 33.1%) and interacting with their cat(s) at home after an appointment was very easy (52.9%). Nearly half of cat caregivers indicated they were very satisfied (47.5%) with their past experience(s) using in-clinic veterinary visits for their cat(s), and over one-third were somewhat satisfied (35.5%).

When asked about their clinic’s COVID-19 examination procedures, the majority stated their clinic offered curbside pickup (77.0%), although some allowed caregivers into the clinic (32.0%) and/or offered telemedicine options, such as phone consultation or emailing (5.3%), video telemedicine (1.7%) or other modalities (7.8%).

### Video telemedicine perceptions

The majority of participants indicated having used a video conferencing platform before (90.1%) and were very comfortable (63.9%) using these platforms (Table 3). Most had not used video telemedicine to access healthcare for their cat(s) (97.3%) or another pet (96.5%), although most indicated using video telemedicine for their own healthcare (72.2%). Over half of the individuals who had used telemedicine for their own healthcare reported being very satisfied with this experience (53.6%).

**Table 3:** Descriptive information on US cat caregivers’ (n = 1254) use of video telemedicine

Variable	Category	n (%)
Used a video conferencing platform before (n = 1254)	Yes	1130 (90.1)
	No	124 (9.9)
Comfort with using video conferencing platforms (n = 1254)	Very comfortable	801 (63.9)
	Somewhat comfortable	283 (22.6)
	Neutral	87 (6.9)
	Somewhat uncomfortable	53 (4.2)
	Very uncomfortable	30 (2.4)
Used video telemedicine for cat(s) (n = 1254)	Yes	34 (2.7)
	No	1220 (97.3)
Used video telemedicine for other pets (not cats) (n = 316)	Yes	11 (3.5)
	No	305 (96.5)
Used video telemedicine for self (n = 1254)	Yes	906 (72.2)
	No	348 (27.8)
Video telemedicine technology utilization (n = 907)	Both computer and phone	553 (61.0)
	Only computer	237 (26.1)
	Only phone	117 (12.9)
Perceived ease of handling their cats during a video telemedicine appointment* (n = 1220)^†^	Very easy	502 (41.1)
	Somewhat easy	457 (37.5)
	Neither easy nor difficult	184 (15.1)
	Somewhat difficult	320 (26.2)
	Very difficult	76 (6.2)
	Not sure	37 (3.0)
Perceived ease of interacting with their cats after a video telemedicine appointment* (n = 1220)^†^	Very easy	912 (74.8)
	Somewhat easy	208 (17.0)
	Neither easy nor difficult	121 (9.9)
	Somewhat difficult	48 (3.9)
	Very difficult	17 (1.4)
	Not sure	34 (2.8)
Accessing the necessary technology for a video appointment (n = 1254)	Very easy	1089 (86.8)
	Somewhat easy	104 (8.3)
	Neither easy nor difficult	38 (3.0)
	Somewhat difficult	15 (1.2)
	Very difficult	8 (0.6)
Ensuring reliable Internet (n = 1254)	Very easy	988 (78.8)
	Somewhat easy	207 (16.5)
	Neither easy nor difficult	32 (2.6)
	Somewhat difficult	24 (1.9)
	Very difficult	3 (0.2)
Website or application use (n = 1254)	Very easy	970 (77.4)
	Somewhat easy	196 (15.6)
	Neither easy nor difficult	53 (4.2)
	Somewhat difficult	31 (2.5)
	Very difficult	4 (0.3)
Webcam or phone camera set-up, positioning and use (n = 1254)	Very easy	831 (66.3)
	Somewhat easy	262 (20.9)
	Neither easy nor difficult	84 (6.7)
	Somewhat difficult	66 (5.3)
	Very difficult	11 (0.9)
Satisfaction using video telemedicine for own healthcare (n = 906)	Very satisfied	486 (53.6)
	Somewhat satisfied	264 (29.1)
	Neither satisfied nor dissatisfied	74 (8.2)
	Somewhat dissatisfied	49 (5.4)
	Very dissatisfied	33 (3.6)
Asked veterinarian about utilizing video telemedicine for one of their pets (n = 1254)	Yes	38 (3.0)
	No	1216 (97.0)
Veterinarian/veterinary staff mentioned using video telemedicine for cat (n = 1254)	Yes	33 (2.6)
	No	1221 (97.4)
Communication the clinic offers* (n = 1254)	Phone calls	1075 (85.7)
	Email	759 (60.5)
	Text messaging	492 (39.2)
	Video conferencing	759 (60.5)
	Telemonitoring	7 (0.6)
	None	24 (1.9)
	Not sure	292 (23.3)

* Respondents could select multiple options

† Had not used veterinary video telemedicine previously

Of those respondents who indicated having used video telemedicine to access veterinary care for their cat(s) (n = 34) or another pet (n = 11), most had used it for an initial health check (cat: 50%, other pet: 45.5%) or a recheck for a health concern (cat: 38.2%, other pet: 36.4%) (Table 4). Respondents who had used video telemedicine with a pet or for themselves (n = 907) indicated using both a computer and phone (61.0%), only a computer (26.1%) or only a phone (12.9%). When asked about handling their cat(s) during a video telemedicine visit, respondents (n = 34) indicated it was very easy (47.1%) or somewhat easy (23.5%), and interacting with their cat(s) after the appointment (n = 27) was very easy (66.7%). Over half (52.6%) of those who had used video telemedicine for their cat(s) and/or other pets (n = 38) reported being very satisfied with their experience.

**Table 4:** Descriptive information on US cat caregiver’s past experiences using video telemedicine with their veterinarian

Variable	Category	n (%)
Video telemedicine usage for cat(s) (n = 34)*	Initial health checkup	17 (50.0)
	Health concern recheck	13 (38.2)
	Initial behavior checkup	4 (11.8)
	Behavior problem recheck	5 (14.7)
	Surgical recheck	2 (5.9)
	Other appointment type	13 (38.2)
Video telemedicine usage for other pets (n = 11)*	Initial health checkup	5 (45.5)
	Health concern recheck	4 (36.4)
	Initial behavior checkup	3 (27.3)
	Behavior problem recheck	2 (18.2)
	Surgical recheck	2 (18.2)
	Other appointment type	2 (18.2)
Handling their cat(s) during a video telemedicine visit (n = 34)*	Very easy	16 (47.1)
	Somewhat easy	8 (23.5)
	Neither easy nor difficult	4 (11.8)
	Somewhat difficult	5 (14.7)
	Very difficult	1 (2.9)
	Not sure	5 (14.7)
Interaction with their cat(s) after the appointment (n = 34)*	Very easy	20 (51.2)
	Somewhat easy	10 (25.6)
	Neither easy nor difficult	4 (10.3)
	Somewhat difficult	4 (10.3)
	Very difficult	1 (2.6)
	Not sure	0 (0)
Satisfaction using video telemedicine with their veterinarian (n = 38)	Very satisfiedSomewhat satisfied	20 (52.6)10 (26.3)
	Neither satisfied nor dissatisfied	5 (13.2)
	Somewhat dissatisfied	0 (0)
	Very dissatisfied	3 (7.9)

* Respondents could select multiple options

The majority of participants who had not undertaken a video telemedicine appointment with their cat (1220/1254) rated the perceived ease of handling their cat(s) for a video appointment as very easy (41.1%) or somewhat easy (37.5%), and perceived that interacting with their cat(s) after an appointment would be very easy (74.8%). Accessing the necessary technology, ensuring a reliable internet connection, using the website(s)/applications needed (eg, Zoom, Facetime, etc), and webcam or phone camera set-up and positioning for a video telemedicine appointment was reported to be very easy (technology: 86.8%, internet: 78.8%, website/application: 77.4%, camera set-up: 66.3%).

Most participants (97.0%) indicated they had not asked their veterinarian about using video telemedicine with one of their pets, and neither had their veterinarian/clinic staff mentioned using it (97.4%). Participants who had asked their veterinarian about using telemedicine, and whose clinic staff or veterinarian had mentioned using video telemedicine for their cats (n = 9), indicated their veterinarian or staff primarily had a very positive (44.4%) or somewhat positive (33.3%) attitude towards using it, with a small percentage somewhat negative or not sure (11.1%). Methods of communication offered by participants’ veterinary clinics were mostly phone calls (85.7%), email (60.5%) and video conferencing (60.5%).

### Perceptions of in-clinic and video telemedicine appointments

Cat caregivers were asked to indicate how frequently cats of varying ages should see a veterinarian and they could select all options that applied. Respondents indicated that kittens (aged <12 months) should have veterinary visits more than once a year (64.0%), when a problem arises (36.6%), once a year (32.2%) and/or every 2–3 years (1.4%). For young adult cats (aged 1–6 years), most respondents indicated they should see a veterinarian annually (74.4%), when a problem arises (44.5%), every 2–3 years (10.4%) and/or more than once a year (7.9%). Caregivers reported that mature adult cats (aged 7–10 years) should have annual veterinary visits (62.4%), when a problem arises (47.2%), more than once a year (21.9%) and/or every 2–3 years (8.1%). For senior cats (aged >10 years), participants indicated more than once a year (50.4%), when a problem arises (49.4%), annual visits (37.9%) and/or every 2–3 years (3.7%). Few respondents indicated that never having a veterinary visit was appropriate for a cat at any age (kittens: 0.1%; young adult cats: 0.2%; mature adult cats: 0.4%; senior cats: 0.2%).

Respondents most frequently indicated that in-clinic appointments had a neutral effect on affording veterinary care (36.4%), finding their cat(s) for the appointment (46.2%), addressing transportation challenges (37.8%) and addressing human disability challenges (62.1%) (Table 5). Participants also indicated that using in-clinic visits for finding appointments that worked with their schedule was somewhat unhelpful (28%), neutral (23.4%), somewhat helpful (22%) or very helpful (18.6%). Participants indicated that in-clinic visits were very helpful for diagnosing health conditions (59.3%), developing treatment plans for health conditions (41.9%) and somewhat helpful for diagnosing behavior problems (34.6%) and developing treatment plans for behavior problems (33.0%).

**Table 5:** Descriptive information on how helpful or unhelpful US cat caregivers (n = 1254) felt each appointment type (in-clinic, video telemedicine) was for various aspects of providing health and behavior care for their cat(s)

Variable	Category	In-clinic (%)	Video telemedicine (%)
Affording veterinary care	Very helpful	12.0	19.0
	Somewhat helpful	14.4	44.6
	Neutral	36.4	32.5
	Somewhat unhelpful	24.7	2.0
	Very unhelpful	12.4	1.9
Finding appointments that work with their schedule	Very helpful	18.6	44.0
	Somewhat helpful	22.0	37.7
	Neutral	23.4	15.5
	Somewhat unhelpful	28.0	1.3
	Very unhelpful	8.1	1.5
Finding their cat(s) for the appointment	Very helpful	11.9	36.0
	Somewhat helpful	11.2	26.8
	Neutral	46.2	32.5
	Somewhat unhelpful	20.7	3.1
	Very unhelpful	10.0	1.6
Addressing transportation challenges	Very helpful	10.4	63.4
	Somewhat helpful	8.1	18.2
	Neutral	37.8	16.3
	Somewhat unhelpful	26.4	0.6
	Very unhelpful	17.3	1.6
Addressing human disability challenges	Very helpful	7.6	46.8
	Somewhat helpful	5.3	15.5
	Neutral	62.1	36.0
	Somewhat unhelpful	14.7	0.7
	Very unhelpful	10.4	1.0
Diagnosing health conditions	Very helpful	59.3	7.7
	Somewhat helpful	24.3	33.3
	Neutral	8.6	29.3
	Somewhat unhelpful	5.1	23.7
	Very unhelpful	2.7	6.1
Developing treatment plans for health conditions	Very helpful	41.9	22.6
	Somewhat helpful	32.9	40.7
	Neutral	17.7	27.2
	Somewhat unhelpful	4.9	6.9
	Very unhelpful	2.6	2.6
Diagnosing behavior problems	Very helpful	23.4	18.7
	Somewhat helpful	34.6	39.5
	Neutral	25.0	28.8
	Somewhat unhelpful	13.5	9.8
	Very unhelpful	3.6	3.3
Developing treatment plans for behavior problems	Very helpful	21.3	26.2
	Somewhat helpful	33.0	40.0
	Neutral	30.9	27.0
	Somewhat unhelpful	10.7	4.4
	Very unhelpful	4.1	2.5

Regarding video telemedicine appointments, respondents frequently reported these appointments would be very helpful for finding their cat(s) for the appointment (36.0%), finding appointments that worked with their schedule (44.0%), addressing transportation challenges (63.4%) and addressing human disability challenges (46.8%). Participants also frequently reported video telemedicine appointments as somewhat helpful for affording veterinary care (44.6%), diagnosing health conditions (33.3%), developing treatment plans for health conditions (40.7%), diagnosing behavior problems (39.5%) and developing treatment plans for behavior problems (40.0%).

**Figure 1:**
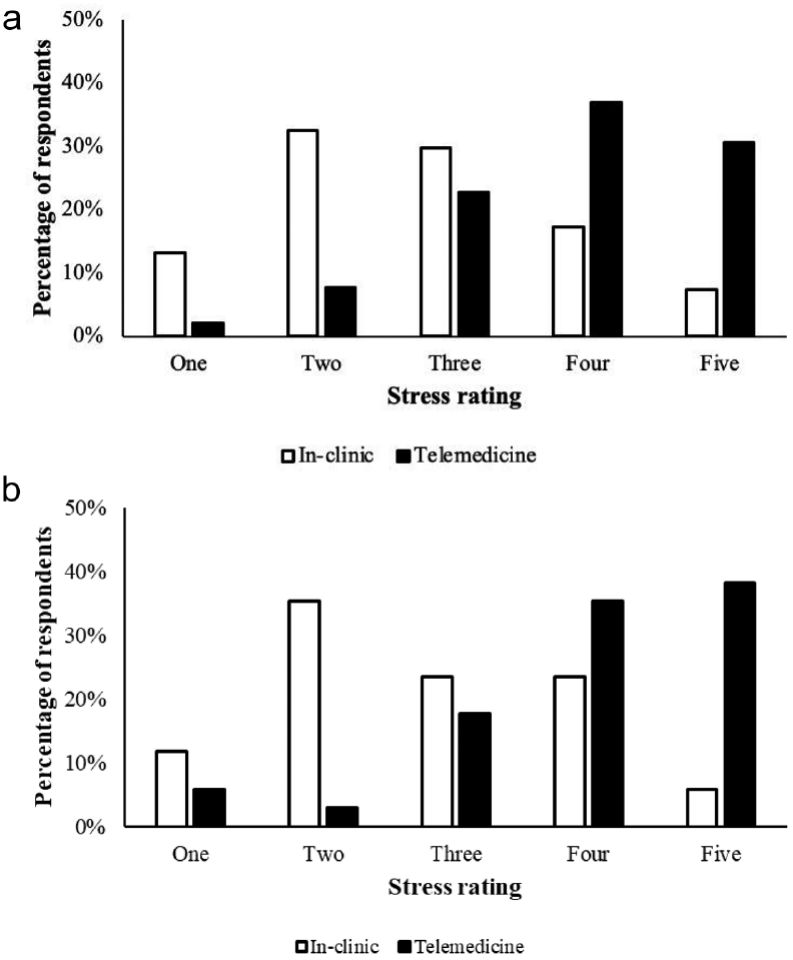
(a) Ratings of respondents who had never used video telemedicine with their cat before (n = 1220), of their own stress level during a video telemedicine vs in-clinic appointment (Wilcoxon signed-rank test, *P* <0.0001). A 5-point Likert scale was used (1 = most stressed, 5 = least stressed). (b) Ratings of respondents who had used video telemedicine with their cat before (n = 34), of their own stress level during a video telemedicine vs in-clinic appointment (Wilcoxon signed-rank test, *P* = 0.0002). A 5-point Likert scale was used (1 = most stressed, 5 = least stressed)

**Figure 2:**
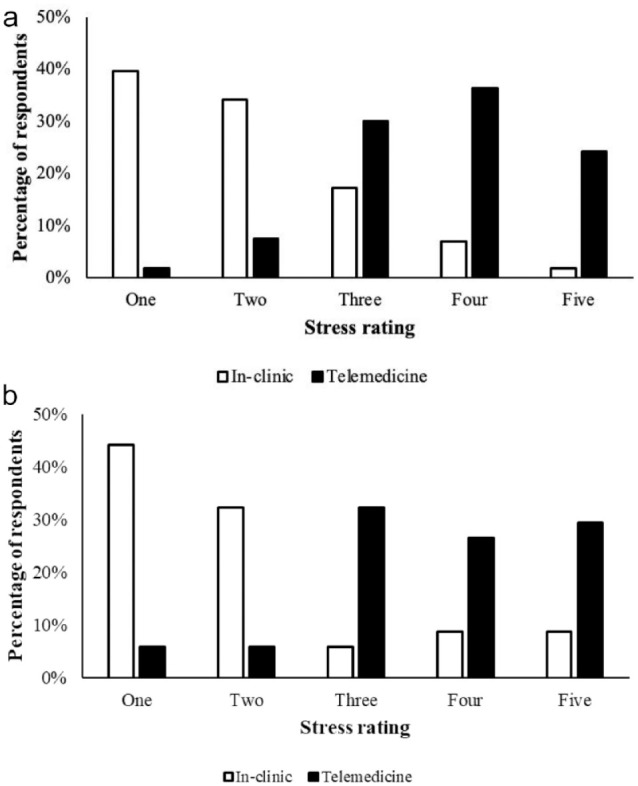
(a) Ratings of respondents who had never used video telemedicine with their cat before (n = 1220), of their cat’s stress level during a video telemedicine vs in-clinic appointment (Wilcoxon signed-rank test, *P* <0.0001). A 5-point Likert scale was used (1 = most stressed, 5 = least stressed). (b) Ratings of respondents who had used video telemedicine with their cat before (n = 34), of their cat’s stress level during a video telemedicine vs in-clinic appointment (Wilcoxon signed-rank test, *P* <0.0001). A 5-point Likert scale was used (1 = most stressed, 5 = least stressed)

Differences were detected between ratings of video telemedicine vs in-clinic appointments for owner stress (used telemedicine with cat: n = 34, *P* = 0.0002; never used telemedicine with cat: n = 1220, *P* <0.0001) and cat stress (used telemedicine with cat: *P* <0.0001, never used telemedicine with cat: *P* <0.0001). Most caregivers who had (n = 34) and had not (n = 1220) used video telemedicine with their cat(s) previously, ranked their own stress levels (1 = most stressed, 5 = least stressed) during a video telemedicine appointment as 4 (used telemedicine with cat: 26.5%, never used telemedicine with cat: 36.9%) or 5 (used telemedicine with cat: 29.4%, never used telemedicine with cat: 30.7%), while most ratings of their stress levels during an in-clinic appointment were 2 (used telemedicine with cat: 35.3%, never used telemedicine with cat: 32.5%), 3 (used telemedicine with cat: 23.5%, never used telemedicine with cat: 29.6%) or 4 (used telemedicine with cat: 23.5%, never used telemedicine with cat: 17.3%) (Figure 1). Most ratings of their cat’s stress levels during a video telemedicine appointment were 3 (used telemedicine with cat: 17.6%, never used telemedicine with cat: 30.1%), 4 (used telemedicine with cat: 35.3%, never used telemedicine with cat: 36.3%) or 5 (used telemedicine with cat: 38.2%, never used telemedicine with cat: 24.3%),while most ratings of their cat’s stress levels during an in-clinic appointment were 1 (used telemedicine with cat: 44.1%, never used telemedicine with cat: 39.7%) or 2 (used telemedicine with cat: 32.4%, never used telemedicine with cat: 34.1%) (Figure 2).

**Figure 3:**
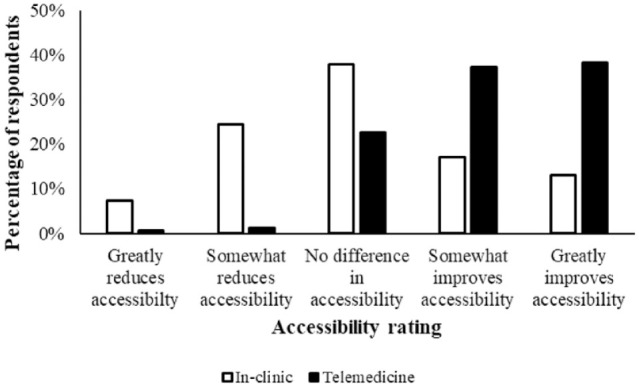
Respondents’ ratings of their access to veterinary care when using a video telemedicine vs in-clinic appointment (n = 1254) (Wilcoxon signed-rank test, *P* <0.0001)

There was a difference in participant ratings of the impact that video telemedicine vs in-clinic appointments had on their access to veterinary care for their cat (n = 1254, *P* <0.0001). Most caregivers ranked video telemedicine as somewhat improving (37.2%) or greatly improving (38.3%) their access to veterinary care, while in-clinic appointments were frequently rated as having no difference (37.9%) or somewhat reducing (24.4%) their access to veterinary care (Figure 3).

### Video telemedicine perceptions

Participants were also asked to rate their interest in using video telemedicine with their cat in the future. Most stated they would be very interested (48.2%) or somewhat interested (37.5%), while others were neutral (5.8%), somewhat uninterested (5.7%) or very uninterested (2.9%). When asked how much they would be willing to pay for a video telemedicine visit, most respondents indicated they would be willing to pay a little less (51.3%), while others indicated much less (27.1%), about the same (18.6%), not willing to pay for this service (1.6%), willing to pay a little more (1.0%) or much more (0.4%) than an in-clinic appointment. The majority of participants (98%) indicated they would be willing to submit videos or photos of their pet to their veterinarian before or during a video telemedicine visit to supplement the appointment.

### Logistic regression results: interest (yes/no) in using video telemedicine

Participants aged 18–29 years had lower odds of being interested in using video telemedicine than those aged 50–59 years (OR 0.16, *P* = 0.0106), and individuals who reported a drive time of 30 mins to under 1 h had greater odds of being interested in video telemedicine than those with a drive time of less than 30 mins (OR 6.35, *P* = 0.0132) (Table 6). Caregivers reporting higher stress levels at the veterinary clinic had greater odds of being interested in using telemedicine than those who found clinic visits less stressful; those who perceived lower levels of stress for themselves during a video telemedicine visit had greater odds of being interested in telemedicine than those who perceived it to be stressful. Caregivers who perceived telemedicine as having no effect on or somewhat reducing their access to veterinary care were less interested in using it than those who expected it to somewhat or greatly improve their access to care. ORs and significance levels for all significant effects are presented in Table 6.

**Table 6:** Logistic regression results showing factors associated with participant interest (yes/no) in using video telemedicine with their cat (n = 1181)

Explanatory variables	Category	OR (95% CI)	*P* value
Age (years)	50–59 (ref)	–	–
	18–29	0.16 (0.035–0.77)	0.01
Travel time to veterinary clinic	<30 mins (ref)	–	–
	30 mins to <1 h	6.35 (1.32–30.46)	0.01
Satisfaction with telemedicine for self	Neither satisfied nor dissatisfied (ref)	–	–
	Very satisfied	5.99 (1.63–22.04)	0.001
	NA (never used)	3.81 (1.10–13.17)	0.03
Owner stress at the clinic	5 (ref)	–	–
(1 = very stressed, 5 = not stressed)	1	6.55 (1.30–32.93)	0.013
	2	6.40 (1.89–21.73)	0.0003
	3	4.39 (1.42–13.63)	0.003
	4	5.01 (1.48–16.92)	0.003
Perceived owner stress when using telemedicine (1 = very stressed, 5 = not stressed)	1 (ref)45	–16.16 (2.2–118.87)8.57 (1.15–63.69)	–0.0010.03
	2 (ref)	–	–
	3	4.03 (1.31–12.38)	0.006
	4	13.72 (3.86–47.62)	<.0001
	5	7.27 (2.02–26.18)	0.0001
	3 (ref)	–	–
	4	3.41 (1.14–10.19)	0.02
Accessibility ratings for telemedicine	Greatly improves accessibility (ref)	–	–
	No difference in accessibility	0.122 (0.04–0.37)	<.0001
	Somewhat reduces accessibility	0.03 (0.003–60.33)	0.001
	Somewhat improves accessibility (ref)	–	–
	No difference in accessibility	0.3 (0.13–0.67)	0.0004
	Somewhat reduces accessibility	0.08 (0.01–0.7)	0.01
Willingness to pay for telemedicine	A little less (ref)	–	–
	Not willing to pay	0.03 (0.002–0.44)	0.003
	Much less	0.32 (0.14–0.74)	0.002
	About the same (ref)	–	–
	Not willing to pay	0.04 (0.003–0.67)	0.01

Only significant findings are listed for brevity. ORs >1 indicate greater odds of interest than the referent (ref) category, while ORs <1 indicate lower odds of interest than the referent category

CI = confidence interval; OR = odds ratio

### Appointment preferences for in-clinic and video telemedicine appointments

Finally, participants were asked to choose the appointment type they would prefer for a variety of common behavior (ie, excessive vocalizations, separation anxiety) and health (ie, dental disease, surgery rechecks, vaccinations) issues. Overall, participants indicated that video telemedicine appointments were generally preferred for behavior concerns (Table 7), while in-clinic appointments were largely preferred for health concerns (Table 8).

**Table 7:** US cat caregivers’ (n = 1254) appointment preferences for common cat behavioral concerns

Behavior problem	In-clinic	Video telemedicine	No preference
Aggression	20.3	62.4	17.4
Excess vocalizations	18.5	60.6	20.9
Destructive behaviors	9.9	69.7	20.4
Eating disorders	38.6	43.9	17.5
Excess nighttime activity	9.3	64.4	26.3
Fears/phobias	9.6	71.8	18.7
Separation anxiety	8.9	73.0	18.2
Unwanted behaviors	5.5	72.9	21.6
Damaging repetitive behaviors	30.5	53.3	16.2
Follow-up, behavior	6.4	78.5	15.1
Help with chronic behavior at home	14.8	69.6	15.6

Data are %

**Table 8:** US cat caregivers’ (n = 1254) appointment preferences for common cat health concerns

Health concern	In-clinic	Video telemedicine	No preference
Urinating/defecating outside litter box	42.0	41.9	16.1
Dental disease	89.9	5.7	4.5
Obesity	26.9	57.8	15.3
Eye disorders	76.2	18.0	5.8
External parasites	48.6	39.5	12.0
Renal disease	86.4	6.6	6.9
Refill medications	8.1	82.1	9.9
Hyperthyroid disease	77.0	13.1	9.9
Internal parasites	75.4	17.8	6.8
Diabetes mellitus	79.3	11.1	9.6
Osteoarthritis	64.9	24.2	10.8
Gastrointestinal disorders	74.4	17.1	8.5
Dermatology concerns	68.2	23.9	7.9
Non-obstructive urinary disease	68.3	22.2	9.6
Obstructive urinary disease	92.5	3.1	4.4
Respiratory concerns	87.6	8.1	4.4
Surgery rechecks	70.6	23.2	6.2
Routine vaccinations	92.7	4.7	2.6
Follow-up, health	19.1	65.6	15.3
Help with chronic health at home	18.5	69.7	11.8
Urgent health issue	86.0	8.9	5.1

Data are %

## Discussion

The study results indicate that many US cat caregivers view veterinary video telemedicine as a way to reduce stress for themselves and their cats, and improve their access to veterinary care. The results suggest that from their perspective, video telemedicine may be useful to overcome some barriers to accessing veterinary care. For example, many participants indicated that video telemedicine is ‘very helpful’ for resolving challenges related to transportation and caregiver disability. Other research has examined pet owner attitudes on the use of telemedicine, with results in line with the current study suggesting that this technology may help increase their access to care and is viewed as beneficial.^21,25,27,29^ A survey of veterinarians in the UK suggested that the benefit of reducing stress for cats and reducing the need for travel is also recognized by veterinarians as an advantage of telemedicine.^30^ Although the benefits of using telemedicine are recognized by both veterinarians and caregivers, our results and other research suggest that many pet owners have never used it.^15,25,27,29^ This is not surprising, given that video telemedicine must be provided as an option by the veterinary team, and research suggests veterinarians may be hesitant to offer video telemedicine, with some planning to decrease or discontinue use after the COVID-19 pandemic.^14,15^ However, it is possible that more research demonstrating the benefits of veterinary video telemedicine may help increase its adoption by practitioners. In addition to cat caregivers’ limited experience with veterinary video telemedicine, a study of pet caregiver attitudes in Denmark, the UK and Austria suggested that the majority of cat caregivers who had not used the technology were ‘not sure’ or would ‘not’ use it in the future.^29^ This was not in line with our findings, as 86% of our study participants indicated they were interested in using it with their cats in the future. This discrepancy may reflect differing care-related attitudes between cat caregivers in the USA and those in the UK and Europe, something that has been observed in other issues of cat care, including provision of outdoor access, which is encouraged in the UK and Europe, but discouraged in the USA.^31,32^

Interestingly, almost all of our participants had not asked their veterinarian about video telemedicine nor had it been mentioned to them by their veterinarian or clinic staff, although close to two-thirds indicated their clinic offered video conferencing. It is possible these visits are only used to communicate with caregivers, not to perform synchronous telemedicine appointments with their pets, including clinical assessment, or that veterinarians are reticent to encourage this use of telemedicine. It is conceivable that for issues requiring more than an email or phone chat, veterinarians prefer to see animals in the clinic, because they are unable to perform a complete physical examination over video, and are concerned about misdiagnoses, communication and technical difficulties.^22,30^ These challenges notwithstanding, video telemedicine appointments may still provide improved quality of care for a pet that cannot be brought into the clinic in a timely manner, and for issues that phone and email communications are insufficient to address. Bishop et al^14^ theorize that decreased monetary compensation for veterinarians when using video telemedicine may be another factor discouraging use. A 2016 survey found most veterinarians never charged for consultations over Skype, which may explain this concern.^33^ However, most of our respondents were willing to pay at least a ‘little less’ for these visits than in-clinic appointments. While this does partially support compensation concerns, under one-third of respondents were willing to pay ‘much less’ for video appointments, and fewer than 2% were unwilling to pay anything at all, suggesting many caregivers do recognize the value of a veterinary video telemedicine visit.

Our study results suggest that cat caregivers and veterinarians may share similar preferences for appointment type (telemedicine vs in-clinic) for health and behavioral concerns. Veterinarians report using telemedicine for dermatological concerns and behavioral issues, as well as follow-ups with ongoing cases, postoperative check-ups, triage and gastrointestinal issues.^14,16^ This usage appears to be in line with industry recommendations.^13^ Participants in the current study indicated preferred use of video telemedicine for a variety of behavioral concerns (ie, separation-related anxiety, dealing with unwanted behaviors, aggression-related problems, follow-up appointments) and some health concerns (ie, follow-up appointments, medication refills, obesity-related concerns and managing chronic health conditions at home). These results again parallel the recommended appointment types for telemedicine,^13^ while also suggesting that cat caregivers recognize the importance of in-clinic appointments for health concerns.

The present study has some limitations. These include almost 80% of survey respondents indicating they had taken their cat to the veterinarian in the past year. Given that past research suggests 43–60% of US cat caregivers take their cat to a veterinarian on a routine basis,^4,5,34^ our results may be impacted by social desirability bias, or study participants valuing cat healthcare more than the average caregiver. In addition, a large population of our participants resided in California, likely because this study originated in California. Most respondents were women without dependents or pets besides cats, which may not reflect many cat owners. Other cat caregiver survey research has shown similar demographic trends,^35–38^ and research suggests that more women tend to participate in online research than men.^39^ Most of our respondents also placed themselves on the upper half of our socioeconomic ladder, indicating feeling above average with regard to socioeconomic status in their communities. Given these demographics, our sample population and thus results may be somewhat homogeneous and biased towards cat-only households. Since recruitment was carried out online via social media groups and news articles, our sample is also likely biased towards individuals who are comfortable with computer and internet use. The small number of caregivers who had used video telemedicine for their cat previously also represents a limitation, as the majority of our sample had never experienced this; therefore, their feelings towards veterinary telemedicine would be expected to reflect perceptions, rather than actual lived experiences, and some of the practical considerations of using the technology may not have been considered. Finally, most participants were from urban and suburban areas; therefore, further research should focus on reaching rural and underserved populations who may benefit from veterinary video telemedicine.

## Conclusions

Overall, the current study results suggest that from the US cat caregiver perspective, incorporating video telemedicine may improve caregiver access to veterinary care, and reduce stress for both cats and caregivers. This is particularly important, given that many cats do not receive veterinary care on a routine basis, thus cats and their caregivers represent an important population that could benefit from the implementation of video telemedicine in veterinary care. Based on participant-reported appointment preferences, cat caregivers are interested in using video telemedicine for most behavioral concerns, some health-related concerns including obesity, medication refills, help managing chronic cat health problems at home and follow-up visits. More research is needed to assess the efficacy and feasibility of using video telemedicine for addressing health and behavior concerns of interest to both veterinarians and cat caregivers.

## Supplemental Material

Table State of residence for US cat caregiver questionnaire respondents (n = 1254)

Supplemental Material Owner attitudes towards use of veterinary video telemedicine
