# Supplementary material for: US cat caregivers’ attitudes on veterinary video telemedicine
Source: J Feline Med Surg. 2024 Aug 9;26(8):1098612X241249623. doi: 10.1177/1098612X241249623 (PMC11418615; doi:10.1177/1098612X241249623)
Supplement: Table [file sj-docx-1-jfm-10.1177_1098612X241249623.docx]

**Supplementary Materials 2:** State of residence for US cat caregiver questionnaire respondents (n=1254).

| **Variable** | **Category** | **No. (%) of respondents** | |  |
| --- | --- | --- | --- | --- |
| State | Alabama | | 6 (0.5 %) | |
|  | Alaska | | 5 (0.4 %) | |
|  | Arizona | | 32 (2.6 %) | |
|  | Arkansas | | 6 (0.5 %) | |
|  | California | | 302 (24.1 %) | |
|  | Colorado | | 40 (3.2 %) | |
|  | Connecticut | | 17 (1.4 %) | |
|  | Delaware | | 3 (0.2 %) | |
|  | Florida | | 59 (4.7 %) | |
|  | Georgia | | 25 (2.0 %) | |
|  | Hawaii | | 7 (0.6 %) | |
|  | Idaho | | 5 (0.4 %) | |
|  | Illinois | | 43 (3.4 %) | |
|  | Indiana | | 21 (1.7 %) | |
|  | Iowa | | 7 (0.6 %) | |
|  | Kansas | | 7 (0.6 %) | |
|  | Kentucky | | 16 (1.3 %) | |
|  | Louisiana | | 4 (0.3 %) | |
|  | Maine | | 6 (0.5 %) | |
|  | Maryland | | 45 (3.6 %) | |
|  | Massachusetts | | 33 (2.6 %) | |
|  | Michigan | | 29 (2.3 %) | |
|  | Minnesota | | 14 (1.1 %) | |
|  | Mississippi | | 1 (0.1 %) | |
|  | Missouri | | 11 (0.9 %) | |
|  | Montana | | 4 (0.3 %) | |
|  | Nebraska | | 8 (0.6 %) | |
|  | Nevada | | 10 (0.8 %) | |
|  | New Hampshire | | 6 (0.5 %) | |
|  | New Jersey | | 33 (2.6 %) | |
|  | New Mexico | | 13 (1.0 %) | |
|  | New York | | 56 (4.5 %) | |

| **Variable** | **Category** | **No. (%) of respondents** |
| --- | --- | --- |
| State, cont. | North Carolina | 36 (2.9 %) |
|  | North Dakota | 2 (0.2 %) |
|  | Ohio | 34 (2.7 %) |
|  | Oklahoma | 7 (0.6 %) |
|  | Oregon | 34 (2.7 %) |
|  | Pennsylvania | 52 (4.1 %) |
|  | Rhode Island | 3 (0.2 %) |
|  | South Carolina | 6 (0.5 %) |
|  | South Dakota | 3 (0.2 %) |
|  | Tennessee | 18 (1.4 %) |
|  | Texas | 48 (3.8 %) |
|  | Utah | 9 (0.7 %) |
|  | Vermont | 4 (0.3 %) |
|  | Virginia | 38 (3.0 %) |
|  | Washington | 55 (4.4 %) |
|  | West Virginia | 7 (0.6 %) |
|  | Wisconsin | 23 (1.8 %) |
|  | Wyoming | 1 (0.1 %) |
