# Supplementary material for: US cat caregivers’ attitudes on veterinary video telemedicine
Source: J Feline Med Surg. 2024 Aug 9;26(8):1098612X241249623. doi: 10.1177/1098612X241249623 (PMC11418615; doi:10.1177/1098612X241249623)
Supplement: Supplemental Material [file sj-docx-2-jfm-10.1177_1098612X241249623.docx]

Owner attitudes towards use of veterinary video telemedicine

Start of Block: Consent Form

**Cat Caretaker Attitudes Toward Use of Veterinary Video Telemedicine**

**Introduction**
You are invited to join a research study conducted by Dr. Carly Moody (Assistant Professor, Dept of Animal Science, UC Davis), and Grace Boone, MS (Assistant Specialist) from the University of California, Davis.

No prior experience with telemedicine is required to participate in this survey.

There are approximately 94.2 million pet cats living in the US, however, many cat caregivers do not take their cat to the veterinarian for annual health and behavior care. Veterinary visits represent an opportunity to detect and address health and behavior concerns.

**Purpose**
The purpose of this study is to investigate cat caregiver attitudes toward using video telemedicine for accessing veterinary health and behavior care for their cats. There is no direct benefit to you from taking part in this study. Study results aim to inform how best to integrate video telemedicine into veterinary care for companion cats.

The risks of this research are minimal. Some of the questions might make you feel uncomfortable or upset. You do not have to answer any of the questions you do not want to answer.

**Participation and Withdrawal**
Participation in this research is completely voluntary.
Participants must be:

- At least 18 years of age
- Currently residing within the United States
- The current primary caregiver of at least one companion cat

Study participation includes this anonymous online survey, which we estimate will take about 15 minutes to complete.

**Benefit to Participant**
There are no direct benefits to participating in the study, however cat health and behavior care communities, as well as cat caregivers may indirectly benefit from this research.

**Confidentiality**
As with all research, there is a chance that confidentiality may be compromised; however, we are taking precautions to minimize this risk. Your responses to the survey will be anonymous and will be kept in password-protected cloud storage.

**Compensation**
You will not be paid for taking part in this study.

**Rights**
Participation in research is completely voluntary. You are free to decline to take part in this study and you may withdraw at any time without consequence. However, please be aware that once your responses have been submitted, it may not be possible to remove them from the data set since they are not linked with your identity.

If you have any questions about this research, please contact the investigator Dr. Carly Moody at cmoody@ucdavis.edu.

Please save or print this page now if you would like a copy for your records. 

**Note:** Your responses for this survey will automatically be saved as you go. You can go back if needed.

**If you have read the above information and agree to take part in the research, please click on the arrow button below.**

End of Block: Consent Form

Start of Block: Inclusion/Exclusion Criteria

1: Do you live within the United States?

- Yes
- No

Skip To: End of Survey If Do you live within the United States? = No

| Page Break |  |
| --- | --- |

2: What state do you live in?

Please select your state from the list:

▼ Alabama ... Wyoming

| Page Break |  |
| --- | --- |

3: How many **cats** are you the primary caretaker for?

- One
- Two
- Three
- Four
- Five or more
- None

Skip To: End of Survey If How many cats are you the primary caretaker for? = None

4: What companion animals, other than cats, are you the primary caretaker for?
 *(please choose all that apply)*

- Dogs
- Other (i.e. small caged mammals, caged reptiles, birds)
- None

| Page Break |  |
| --- | --- |

5: What is your age?

- Under 18
- 18 - 29
- 30 - 39
- 40 - 49
- 50 - 59
- 60 - 69
- 70 or older
- Prefer not to say, but over 18 years of age

Skip To: End of Survey If What is your age? = Under 18

End of Block: Inclusion/Exclusion Criteria

Start of Block: Human Demographics

6: Which gender identity do you most identify with?

- Man
- Woman
- Non-binary, third gender, or other
- Prefer not to say

7: Which option best describes where you currently live?

- Urban area (city center or metropolis)
- Suburban (residential area on the outskirts of a city)
- Rural (settled place outside of a city)

| Page Break |  |
| --- | --- |

8: Do you have dependents (e.g. children, others that rely on you for care) in your household?

- No
- Yes

| Page Break |  |
| --- | --- |

9: **Socioeconomic Ladder:**
This ladder represents where people stand in their local communities. At the top of the ladder are people who are well off – those who have the most money, education, and highest paying jobs. At the bottom are people who are

not well off – those who have the least money, education, and lower paying jobs. The higher up you are on this ladder, the closer you are to the people at the very top; the lower you are, the closer you are to the people at the very bottom.

 **Where would you place yourself on this ladder?**
 Please choose the number corresponding to the step/level you think you currently stand on, relative to other people *in your community*.

▼ 1 ... 10

| Page Break |  |
| --- | --- |

10: How many total years of **volunteer or work experience** do you have with cats, NOT including as a cat owner? (e.g. animal shelter volunteer, pet-sitter, trainer, veterinary staff)

- I have **never** worked/volunteered with cats
- Less than 1 year
- 1-5 years
- 6-10 years
- More than 10 years

Display This Question:

If How many total years of volunteer or work experience do you have with cats, NOT including as a ca... != I have <strong>never</strong> worked/volunteered with cats

11: In what capacity did or do you work with cats?
 *(please choose all that apply)*

- Behaviorist
- Groomer
- Pet-sitter
- Animal shelter staff/volunteer
- Trainer
- Working in a veterinary clinic (e.g., Veterinarian, Veterinary staff, Veterinary student)
- Other (please specify) __________________________________________________

End of Block: Human Demographics

Start of Block: Owner Experience: In-Clinic

The following questions will focus on your previous experiences with **in-clinic** veterinary appointments.

| Page Break |  |
| --- | --- |

12: Approximately when was your last veterinary visit with any of your cats?

- Within the past year
- 1-2 years ago
- 3-5 years ago
- More than 5 years ago
- Never

| Page Break |  |
| --- | --- |

13: Has a veterinarian ever prescribed calming or sedating medications that you have to give **at home** (e.g. Gabapentin) before bringing your cat(s) to the veterinary clinic?

- Yes
- No
- Not sure

14: Do any of your cat(s) have to be sedated **at the veterinary clinic** for an exam
(i.e., due to handling difficulties/aggression), not including surgery or painful procedures?

- Often
- Sometimes
- Rarely
- Never
- Not sure

| Page Break |  |
| --- | --- |

15: During the COVID-19 pandemic, my veterinary clinic has:
*(please select all that apply)*

- Allowed me to come into the clinic with my cat(s) as normal
- Offered curbside appointments only (I am not allowed into the clinic with my cat/cats)
- Offered video telemedicine (video conferencing with my veterinarian over a video platform such as Zoom)
- Offered other telemedicine appointments (e.g. phone consultation, emailing, texting)
- Other (please specify) __________________________________________________

| Page Break |  |
| --- | --- |

16: How long does it usually take for you to travel to your veterinary clinic (one-way)?

- Less than 30 minutes
- 30 minutes - under 1 hour
- 1 hour - 1.5 hours
- More than 1.5 hours

17: Do you implement any of the following before or during the car ride to prepare your cat(s) for their veterinary visit?
*(please choose all that apply)*

- Spray the carrier with a pheromone spray (i.e., Feliway)
- Leave the carrier out all the time
- Take the carrier out before the appointment (i.e., hours or days before appointment)
- Play soothing music in the car
- Cover the carrier with a towel during travel
- Other (please describe) __________________________________________________
- None of the above

| Page Break |  |
| --- | --- |

18: In your experience, during an **in-clinic** veterinary visit for your cat(s), how easy or difficult is it: **(Please select all that apply if you have more than 1 cat)**

 *For example, if you have one cat that is very difficult to handle, and another that is very easy to handle, select both 'very difficult' and 'very easy'*

|  | Very difficult | Somewhat difficult | Neither easy nor difficult | Somewhat easy | Very easy | Not sure |
| --- | --- | --- | --- | --- | --- | --- |
| For your veterinarian to handle your cat(s)? |  |  |  |  |  |  |
| For the veterinary staff to handle your cat(s)? |  |  |  |  |  |  |
| To interact with your cat(s) at home after the appointment? |  |  |  |  |  |  |

| Page Break |  |
| --- | --- |

19:On average, how satisfied are you with your past experience(s) using **in-clinic** veterinary visits for your cat(s)?

- Very satisfied
- Somewhat satisfied
- Neither satisfied nor dissatisfied
- Somewhat dissatisfied
- Very dissatisfied

End of Block: Owner Experience: In-Clinic

Start of Block: Owner Experience: Telemedicine

The following questions will focus on your previous experiences with **video conferencing software and video telemedicine** appointments.

| Page Break |  |
| --- | --- |

20: Have you ever used a video conferencing platform (e.g., Zoom, Facetime, Skype, Microsoft Teams, Google Meet) before?

- No
- Yes

21: How comfortable are you with using video conferencing platforms?

- Very comfortable
- Somewhat comfortable
- Neutral
- Somewhat uncomfortable
- Very uncomfortable

| Page Break |  |
| --- | --- |

VTM Definition **For the following questions, please keep this definition in mind:**

- Video telemedicine = Providing health care and/or behavior care for a pet or person over a distance using video conferencing (e.g. Zoom)

22: Have you ever had a **video telemedicine** veterinary visit for your cat(s)?
(In other words, have you ever used video conferencing software such as Zoom, Facetime, Skype, Microsoft Teams, Google Meet, with your veterinarian?)

- No
- Yes

Display This Question:

If What companion animals, other than cats, are you the primary caretaker for?(please choose all tha... = Dogs

Or What companion animals, other than cats, are you the primary caretaker for?(please choose all tha... = Other (i.e. small caged mammals, caged reptiles, birds)

And What companion animals, other than cats, are you the primary caretaker for?(please choose all tha... != None

22: Have you ever had a **video telemedicine** visit for one of your pets that is not a cat?

- No
- Yes

| Page Break |  |
| --- | --- |

23: Have you ever had a **video telemedicine** appointment **for yourself** with a healthcare provider (i.e., physician, nurse, dermatologist, etc.)?

- Yes
- No

| Page Break |  |
| --- | --- |

Display This Question:

If Have you ever had a video telemedicine veterinary visit for your cat(s)?  (In other words, have y... = Yes

And Have you ever had a video telemedicine visit for one of your pets that is not a cat?  = Yes

Or Have you ever had a video telemedicine appointment for yourself with a healthcare provider (i.e.,... = Yes

24: Have you used a phone, computer/tablet or both for **video telemedicine**?

- Phone
- Computer (i.e., desktop computer, laptop, tablet)
- Both

| Page Break |  |
| --- | --- |

Display This Question:

If Have you ever had a video telemedicine veterinary visit for your cat(s)?  (In other words, have y... = Yes

25: For your **cat(s)**, what types of **video telemedicine** visits have you had?
 *(please check all that apply)*

- Initial visit for a health concern
- Recheck for a health concern
- Surgical recheck
- Initial visit for a behavior problem
- Recheck for a behavior problem
- Other (please specify) __________________________________________________

| Page Break |  |
| --- | --- |

Display This Question:

If Have you ever had a video telemedicine visit for one of your pets that is not a cat?  = Yes

26: For your **pets that are not cats**, what types of **video telemedicine** visits have you had?
 *(please check all that apply)*

- Initial visit for a health concern
- Recheck for a health concern
- Surgical recheck
- Initial visit for a behavior problem
- Recheck for a behavior problem
- Other (please specify) __________________________________________________

| Page Break |  |
| --- | --- |

Display This Question:

If Have you ever had a video telemedicine veterinary visit for your cat(s)?  (In other words, have y... = Yes

27a: In your experience, during a **video telemedicine** visit for your cat(s), how easy or difficult is it: **(Please select all that apply if you have more than 1 cat)**

 *For example, if you have one cat that is very difficult to handle, and another that is very easy to handle, select both 'very difficult' and 'very easy'*

|  | Very difficult | Somewhat difficult | Neither easy nor difficult | Somewhat easy | Very easy | Not sure |
| --- | --- | --- | --- | --- | --- | --- |
| For you to handle your cat(s)? |  |  |  |  |  |  |
| To interact with your cat(s) at home after the appointment? |  |  |  |  |  |  |

| Page Break |  |
| --- | --- |

Display This Question:

If Have you ever had a video telemedicine veterinary visit for your cat(s)?  (In other words, have y... = No

27b: If you were to use **video telemedicine** for your cat(s), how easy or difficult do you think it would be: **(Select all that apply if you have more than 1 cat)**

 *For example, if you have one cat that is very difficult to handle, and another that is very easy to handle, select both 'very difficult' and 'very easy'*

|  | Very difficult | Somewhat difficult | Neither easy nor difficult | Somewhat easy | Very easy | Not sure |
| --- | --- | --- | --- | --- | --- | --- |
| For you to handle your cat(s)? |  |  |  |  |  |  |
| To interact with your cat(s) at home after the appointment? |  |  |  |  |  |  |

| Page Break |  |
| --- | --- |

28: Please rate your ability to access the following, for a veterinary **video telemedicine** appointment:

|  | Very easy | Somewhat easy | Neither easy nor difficult | Somewhat difficult | Very difficult |
| --- | --- | --- | --- | --- | --- |
| Accessing the necessary technology (e.g., computer, tablet, smartphone): |  |  |  |  |  |
| Ensuring reliable internet: |  |  |  |  |  |
| Using the website(s)/app(s) needed (e.g., zoom, facetime, etc): |  |  |  |  |  |
| Webcam (or phone camera) set up, positioning, & use: |  |  |  |  |  |

Display This Question:

If Have you ever had a video telemedicine visit for one of your pets that is not a cat?  = Yes

Or Have you ever had a video telemedicine veterinary visit for your cat(s)?  (In other words, have y... = Yes

29: How satisfied were you with your past experience(s) using **video telemedicine** **with your veterinarian**?

- Very satisfied
- Somewhat satisfied
- Neither satisfied nor dissatisfied
- Somewhat dissatisfied
- Very dissatisfied

Display This Question:

If Have you ever had a video telemedicine appointment for yourself with a healthcare provider (i.e.,... = Yes

30: How satisfied were you with your past experience(s) using **video telemedicine** **for your own healthcare**?

- Very dissatisfied
- Somewhat dissatisfied
- Neither satisfied nor dissatisfied
- Somewhat satisfied
- Very satisfied

31: Have you ever asked your veterinarian about using **video telemedicine** with one of your pets?

- No
- Yes

32: Has your veterinarian or veterinary staff ever mentioned using **video telemedicine** with you and your cat?

- Yes
- No

Display This Question:

If Have you ever asked your veterinarian about using video telemedicine with one of your pets? = Yes

And Has your veterinarian or veterinary staff ever mentioned using video telemedicine with you and yo... = Yes

33: From your perspective, what was your **veterinarian or veterinary staff's attitude** about using **video telemedicine** with you and your cat?

- Very negative
- Somewhat negative
- Neutral
- Somewhat positive
- Very Positive
- Not sure

34: What methods of communication does your veterinary clinic offer?
 *(Please select all that apply)*

- Emailing (includes pictures/videos, medication refills, etc.)
- Video conferencing (i.e., zoom, skype, etc.)
- Phone calls
- Text messaging
- Tele-monitoring: remote monitoring of animal vital signs and behaviors (i.e., wearable technology)
- I am not sure what options my veterinary clinic offers
- None of the above

End of Block: Owner Experience: Telemedicine

Start of Block: Owner Opinions: In-Clinic

The following questions ask for your opinions about **in-clinic** veterinary visits appointments.

| Page Break |  |
| --- | --- |

35: How frequently do you think a cat in the following life stages needs to see a veterinarian? **(select all that apply)**

|  | Once a year | More than once a year | Every 2-3 years | When a health/behavior problem arises | Never |
| --- | --- | --- | --- | --- | --- |
| Kitten (less than 12 months) |  |  |  |  |  |
| Young Adult (1-6 years) |  |  |  |  |  |
| Mature (7-10 years) |  |  |  |  |  |
| Senior (over 10 years) |  |  |  |  |  |

| Page Break |  |
| --- | --- |

36: Would you be willing to submit videos or photos of your pet to your veterinarian before or during an in-clinic visit to supplement the appointment?

- No
- Yes

| Page Break |  |
| --- | --- |

37: Please rate how helpful or unhelpful you feel **in-clinic** appointments are for the following aspects of providing health and behavior care for your cat(s):

|  | Very unhelpful | Somewhat unhelpful | Neutral | Somewhat helpful | Very helpful |
| --- | --- | --- | --- | --- | --- |
| Affording veterinary care |  |  |  |  |  |
| Finding appointments that work with my schedule |  |  |  |  |  |
| Finding my cat(s) for their appointment |  |  |  |  |  |
| Addressing transportation challenges (e.g. cat perception of travel, vehicle availability, etc.) |  |  |  |  |  |
| Addressing human disability challenges |  |  |  |  |  |
| Diagnosing health conditions |  |  |  |  |  |
| Diagnosing behavior problems |  |  |  |  |  |
| Developing treatment plans for health conditions (i.e. translating treatment plans from the hospital to your home) |  |  |  |  |  |
| Developing treatment plans for behavior problems (i.e. coming up with treatment methods that work for you at home) |  |  |  |  |  |

| Page Break |  |
| --- | --- |

38: On average, how stressful is it **for you** to take your cat(s) to the veterinary clinic? Please select the number that corresponds to the stress level.

- 1
- 2
- 3
- 4
- 5


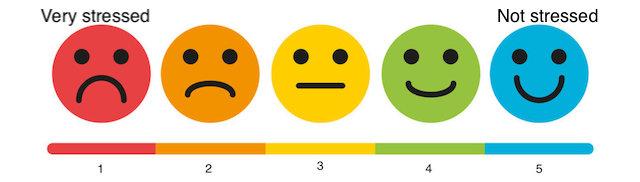


39: On average, how stressful is it **for your cat(s)** to go to the veterinary clinic? Please select the number that corresponds to the stress level.

- 1
- 2
- 3
- 4
- 5


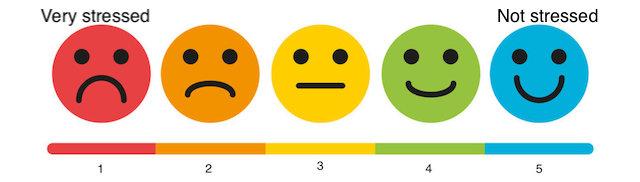


| Page Break |  |
| --- | --- |

40: How does the option of an **in-clinic** appointment impact your accessibility to veterinary care, in **comparison to video telemedicine**?

- Greatly improves accessibility
- Somewhat improves accessibility
- No difference in accessibility
- Somewhat reduces accessibility
- Greatly reduces accessibility

End of Block: Owner Opinions: In-Clinic

Start of Block: Owner Opinions: Telemedicine

The following questions will ask for your opinions about **veterinary video telemedicine** appointments.

| Page Break |  |
| --- | --- |

42: If veterinary **video telemedicine** were available to you, please rate your interest in using it for your cat(s):

- Very interested
- Somewhat interested
- Neutral
- Somewhat uninterested
- Very uninterested

43: In comparison to what you would expect to pay for an **in-clinic visit** (non-emergency), how much are you willing to pay for a **video telemedicine** visit?

- I would not be willing to pay for this service
- Much less
- A little less
- About the same
- A little more
- Much more

44: Would you be willing to submit videos or photos of your pet to your veterinarian before or during a **video telemedicine** visit to supplement the appointment?

- No
- Yes

| Page Break |  |
| --- | --- |

45: Please rate how helpful or unhelpful you feel video telemedicine appointments are for the following aspects of providing health and behavior care for your cat(s):

|  | Very unhelpful | Somewhat unhelpful | Neutral | Somewhat helpful | Very helpful |
| --- | --- | --- | --- | --- | --- |
| Affording veterinary care |  |  |  |  |  |
| Finding appointments that work with my schedule |  |  |  |  |  |
| Finding my cat(s) for their appointment |  |  |  |  |  |
| Addressing transportation challenges (e.g. cat perception of travel, vehicle availability, human mobility/injury, etc.) |  |  |  |  |  |
| Addressing human disability challenges |  |  |  |  |  |
| Diagnosing health conditions |  |  |  |  |  |
| Diagnosing behavior problems |  |  |  |  |  |
| Developing treatment plans for health conditions (i.e. translating treatment plans from the hospital to your home) |  |  |  |  |  |
| Developing treatment plans for behavior problems (i.e. coming up with treatment methods that work for you at home) |  |  |  |  |  |

| Page Break |  |
| --- | --- |

Display This Question:

If Have you ever had a video telemedicine veterinary visit for your cat(s)?  (In other words, have y... = No

46a: How stressful do you think it would be **for your cat** to have a **video telemedicine** appointment with you and your veterinarian? Please select the number that corresponds to the stress level.

- 1
- 2
- 3
- 4
- 5


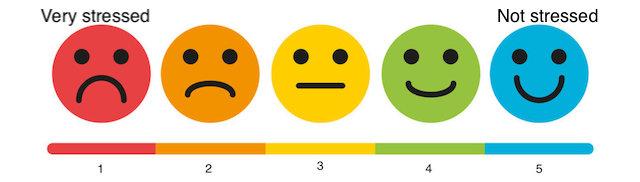


Display This Question:

If Have you ever had a video telemedicine veterinary visit for your cat(s)?  (In other words, have y... = No

47a: How stressful do you think it would be **for you** to have a **video telemedicine** appointment with your cat and your veterinarian? Please select the number that corresponds to the stress level.

- 1
- 2
- 3
- 4
- 5


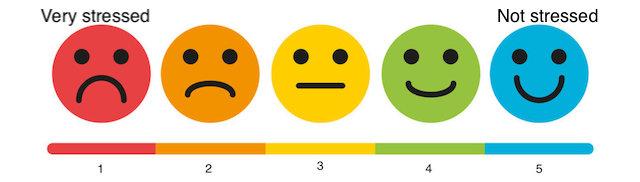


Display This Question:

If Have you ever had a video telemedicine veterinary visit for your cat(s)?  (In other words, have y... = Yes

46b: On average, how stressful is it **for your cat** to have a **video telemedicine** appointment with you and your veterinarian? Please select the number that corresponds to the stress level.

- 1
- 2
- 3
- 4
- 5


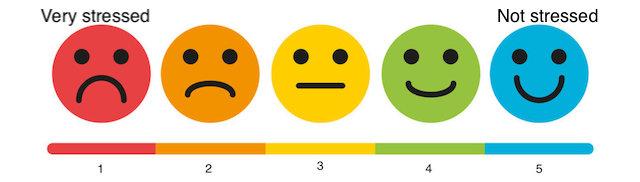


Display This Question:

If Have you ever had a video telemedicine veterinary visit for your cat(s)?  (In other words, have y... = Yes

47b: On average, how stressful is it **for you** to have a **video telemedicine** appointment with your cat and your veterinarian? Please select the number that corresponds to the stress level.

- 1
- 2
- 3
- 4
- 5


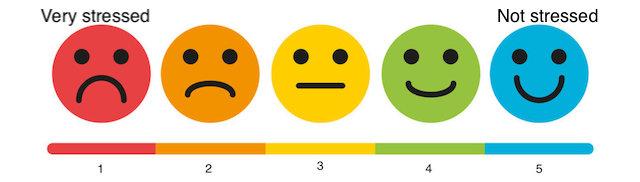


| Page Break |  |
| --- | --- |

8: How does the option of **video telemedicine** impact your accessibility to veterinary care, in **comparison to an in-clinic appointment?**

- Greatly improves accessibility
- Somewhat improves accessibility
- No difference in accessibility
- Somewhat reduces accessibility
- Greatly reduces accessibility

End of Block: Owner Opinions: Telemedicine

Start of Block: Comparisons of in-clinic and telemedicine

The following questions will ask you to choose your preferred appointment type for various common cat health and behavior concerns.

Please answer each question to the best of your ability. If you make a mistake, you can use the back button to return to previous questions.

| Page Break |  |
| --- | --- |

49: Based on your previous experiences, please indicate your preference of appointment type for the following common **cat behavior concerns** with your cat(s):

|  | Appointment Location | | |
| --- | --- | --- | --- |
|  | In-clinic | Video telemedicine | No preference |
| Aggression (e.g., toward people and/or animals) |  |  |  |
| Excessive vocalizations |  |  |  |
| Destructive behaviors (e.g., scratching) |  |  |  |
| Eating disorders (e.g., eating non food items, feces) |  |  |  |
| Excessive nighttime activity |  |  |  |
| Fears/phobias (e.g. noise phobias) |  |  |  |
| Separation anxiety |  |  |  |
| Unwanted behaviors (e.g., jumping on counters, stealing food) |  |  |  |
| Damaging repetitive behaviors (e.g., excessive grooming, pacing) |  |  |  |
| Following up about a behavior concern |  |  |  |
| Help with maintenance of at-home treatments for chronic behavior conditions |  |  |  |
| Urinating/defecating outside the litter box |  |  |  |

| Page Break |  |
| --- | --- |

50: Based on your previous experiences, please indicate your preference of appointment type for the following common **cat health issues** with your cat(s):

|  | Appointment Location | | |
| --- | --- | --- | --- |
|  | In-clinic | Video telemedicine | No preference |
| Dental disease |  |  |  |
| Obesity |  |  |  |
| Eye disorders (e.g., conjunctivitis) |  |  |  |
| External parasites (e.g., fleas) |  |  |  |
| Renal disease |  |  |  |
| Refilling medication prescriptions |  |  |  |
| Hyperthyroidism |  |  |  |
| Internal parasites (e.g., worms) |  |  |  |
| Diabetes Mellitus |  |  |  |
| Osteoarthritis |  |  |  |
| Gastrointestinal disorders (e.g., diarrhea) |  |  |  |
| Dermatological disorders (e.g., skin allergies) |  |  |  |
| Non-obstructive urinary diseases |  |  |  |
| Obstructive urinary diseases |  |  |  |
| Respiratory diseases |  |  |  |
| Surgery re-check |  |  |  |
| Routine vaccinations |  |  |  |
| Follow up appointments |  |  |  |
| Help with maintenance of at-home treatments for chronic health conditions |  |  |  |
| Urgent situation (e.g., medication side effects, toxin exposure/ingestion) |  |  |  |

End of Block: Comparisons of in-clinic and telemedicine

Start of Block: Open-ended box

54: Is there anything else you would like to add about your experiences with in-clinic or video telemedicine for accessing cat health and behavior care?

________________________________________________________________

________________________________________________________________

________________________________________________________________

________________________________________________________________

________________________________________________________________

End of Block: Open-ended box
